# NLSDeconv: an efficient cell-type deconvolution method for spatial transcriptomics data

**DOI:** 10.1093/bioinformatics/btae747

**Published:** 2024-12-20

**Authors:** Yunlu Chen, Feng Ruan, Ji-Ping Wang

**Affiliations:** Department of Statistics and Data Science, Northwestern University, Evanston, IL 60208, United States; Department of Statistics and Data Science, Northwestern University, Evanston, IL 60208, United States; Department of Statistics and Data Science, Northwestern University, Evanston, IL 60208, United States

## Abstract

**Summary:**

Spatial transcriptomics (ST) allows gene expression profiling within intact tissue samples but lacks single-cell resolution. This necessitates computational deconvolution methods to estimate the contributions of distinct cell types. This article introduces NLSDeconv, a novel cell-type deconvolution method based on non-negative least squares, along with an accompanying Python package. Benchmarking against 18 existing deconvolution methods on various ST datasets demonstrates NLSDeconv’s competitive statistical performance and superior computational efficiency.

**Availability and implementation:**

NLSDeconv is freely available at https://github.com/tinachentc/NLSDeconv as a Python package.

## 1 Introduction

Spatial transcriptomics (ST), crowned the Method of the Year in 2020 (Marx 2021), has revolutionized biomedical research by allowing quantification of the mRNA expression of a large number of genes simultaneously in the spatial context of the tissue. The sequencing-based ST technology, while achieving its popularity for spatial profiling of gene expression across larger tissue areas with higher throughput than the image-based ST methods, lacks single-cell resolution. Consequently, the gene expression measured within a spot or a section of the tissue only reflects the average expression of a mixture of cell populations. Understanding the precise cell type composition within each spatial location is crucial for studying developmental biology and cancer biology. Thus, deconvoluting the cell type composition within each spot has become an imperative task in ST research.

Cell-type deconvolution in ST data often utilizes a set of reference gene expression profiles for known cell types, frequently obtained from scRNA-seq studies (Moses and Pachter 2022). Many methods have been developed in recent years, including probabilistic-based methods (Andersson *et al.* 2020, Cable *et al.* 2022, Danaher *et al.* 2022, Kleshchevnikov *et al.* 2022, Lopez *et al.* 2022, Ma and Zhou 2022); non-negative matrix factorization-based methods (Rodriques *et al.* 2019, Dong and Yuan 2021, Elosua-Bayes *et al.* 2021); and deep learning-based methods (Biancalani *et al.* 2021), etc. Some of these methods utilize the spatial information [such as Ma and Zhou (2022)], while many do not (Dong and Yuan 2021, Cable *et al.* 2022, Kleshchevnikov *et al.* 2022). Therefore, in principle many methodologies developed for cell-type deconvolution for bulk RNA-seq data such as Newman *et al.* (2015), Hunt *et al.* (2019), and Li and Wu (2019) can be applied to ST deconvolution problem as well. For excellent reviews of the deconvolution topic in ST data, we refer to Chen *et al.* (2022) and Li *et al.* (2023).

In this article, we propose a novel method named NLSDeconv and a Python package based on the non-negative least squares (NNLS) method for cell-type deconvolution for ST data. We demonstrate the competitive performance of NLSDeconv relative to 18 existing methods by utilizing comprehensive benchmark datasets from both image-based and sequencing-based ST technologies, as well as the compelling efficiency of NLSDeconv.

## 2 Materials and methods

Like many ST cell-type deconvolution methods, NLSDeconv takes two input datasets.

- An ST dataset containing RNA-seq read count for each gene at each spot within the tissue, denoted by **Y** of size $n_{s}\times n_{g}$ where *n_s_* and *n_g_* stand for the number of spots (spatial locations) and number of genes, respectively.
- A single-cell RNA-seq (scRNA-seq) reference dataset, denoted **X**, with dimensions $n_{c}\times n_{g}$, contains the genome-wide expression in read counts of *n_g_* genes collected from *n_c_* cells. Each cell is known to belong to one of *K* distinct cell types (typically $K<<n_{c}$). Each cell type contains multiple biological profiles (i.e. multiple rows of **X**), and their gene expression profiles are defined as prototypes of that cell type in the reference datasets. For example, in the seqFISH3000 dataset, the cell type “iNeuron” is represented by 164 prototypes, meaning 164 rows of **X** correspond to the same cell type “iNeuron,” with each of the 164 rows reflecting a unique gene expression profile. The deconvolution algorithm estimates the proportion of each prototype in each spot using the observed spatial transcriptomic data. The proportion of a cell type is then calculated as the sum of the proportions of all its prototypes. For instance, the overall proportion of the “iNeuron” cell type is the sum of the 164 prototype proportions.
- Here, we assume **X** and **Y** share the same gene dimension *n_g_*, but note that NLSDeconv can adjust for different dimensions through gene selection preprocessing.

The output of NLSDeconv is a cell-type deconvolution matrix $\hat{P}$ with each entry estimating the proportion of a cell *type* (not prototype) within a spot in the tissue.

The fundamental idea behind NLSDeconv is modeling the ST data’s gene read counts at a spot as a *weighted sum* of contributions from each cell prototype present. This is captured by a weight matrix **M** of size $n_{s}\times n_{c}$, where each entry indicates the weight of cell prototypes at a spot. The relationship is formulated by a linear model:

(1) Y=MX+E.

where **E** is a noise matrix of dimensions $n_{s}\times n_{g}$. This linear model captures how various cell prototypes contribute to the observed gene read counts across different spots, with $M_{ij}=0$ indicating no contribution from the *j*^th^ cell prototype at the *i*^th^ spot.

In the field of ST cell-deconvolution, many existing linear models, such as those documented by Rodriques *et al.* (2019), Dong and Yuan (2021), and Elosua-Bayes *et al.* (2021), typically regress **Y** onto the cell type. In contrast, our approach regresses **Y** onto the cell prototype **X**. Our method (see below) essentially builds on top of least squares objectives to solve the linear model, which differs significantly from the method in Biancalani *et al.* (2021). That method employs Kullback–Leibler divergence and cosine distance to learn the matrix **M**, even though it similarly regresses **Y** on the cell prototypes **X**.

To estimate the weight matrix **M** in our method, we employ NNLS (Hastie *et al.* 2009). Our NNLS estimate ${\hat{M}}^{\text{NNLS}}$ of **M** is defined by:

(2) $${\hat{M}}^{\text{NNLS}}=\underset{M\geq 0}{\text{argmin}}\frac{1}{2}||Y-\mathrm{MX}|{|}_{F}^{2}+\frac{\lambda }{2}||M|{|}_{F}^{2}.$$

The Frobenius norm ${\left|\left|\cdot \right|\right|}_{F}$ measures model fidelity, λ≥0 is a ridge regularization parameter, and the constraint M≥0 requires all entries of the matrix **M** to be nonnegative. The NNLS objective is convex, allowing projected gradient descent to converge to its global minimum (Boyd and Vandenberghe 2004). To select the regularization parameter *λ*, we use cross-validation. For each λ≥0, we split the data by genes, fit the model using the training data, and validate its performance on the test data. More precisely, consider splitting the data matrices **X** and **Y** by the genes, i.e. by columns (recalling that each column represents a gene):

$$Y=[Y_{\text{tr}},Y_{\text{test}}],\;\;\;X=[X_{\text{tr}},X_{\text{test}}].$$

Here, $Y_{\text{tr}}$ has dimensions $n_{s}\times n_{g,\text{tr}}$ and $Y_{\text{test}}$ has dimensions $n_{s}\times n_{g,\text{test}}$ with $n_{g}=n_{g,\text{tr}}+n_{g,\text{test}}$. The matrix **X** is split in the same way, with matching column dimensions to their **Y** counterparts. We fit the model on the training data:

$${\hat{M_{\text{tr}}}}^{\text{NNLS}}(\lambda )=\underset{M\geq 0}{\text{argmin}}\frac{1}{2}||Y_{\text{tr}}-MX_{\text{tr}}|{|}_{F}^{2}+\frac{\lambda }{2}||M|{|}_{F}^{2}.$$

The performance is then validated on the test data by:

$$\text{MMSE}(\lambda )=\frac{1}{2n_{g,\text{test}}}||Y_{\text{test}}-{\hat{M_{\text{tr}}}}^{\text{NNLS}}(\lambda )X_{\text{test}}|{|}_{F}^{2}.$$

We select the *λ* that minimizes the MMSE(λ) on the test data. This procedure, here described as a single split, can be directly extended to cross-validation by repeating across multiple folds. In practice, 5-fold cross-validation is often used. To reduce computational burdens, we also suggest using a default λ=0.1 in our package, as empirically it has shown a good performance across a collection of datasets (see Section 4).

The method then calculates the cell-type deconvolution matrix ${\hat{P}}^{\text{NLS}}$ using the weight matrix estimator ${\hat{M}}^{\text{NLS}}$. The proportion of cell type k∈{1,…,K} at spot *i* is calculated as follows:

(3) $${\hat{P}}_{ik}^{\text{NNLS}}=\frac{\sum_{j\in \text{cell}\;\text{type}\;k}{\hat{M}}_{ij}^{\text{NNLS}}}{\sum_{j}{\hat{M}}_{ij}^{\text{NNLS}}},$$

where the numerator is the sum of weights of cell type *k* at spot *i*, and the denominator is the total sum of weights across all cell types at that spot. This results in ${\hat{P}}^{\text{NNLS}}$, an estimator for the cell-type deconvolution matrix.

In practice, computing ${\hat{M}}^{\text{NNLS}}$, consequentially ${\hat{P}}^{\text{NNLS}}$ for large datasets can be challenging due to the complexities of solving the NNLS minimization as described in Equation (2). To enhance computational efficiency, we propose a variant of the NNLS method, called soft-thresholding least squares (SLS). This approach starts with the ordinary least squares (OLS) estimator which has the following closed form without the constraint M≥0 in (2):

(4) $${\hat{M}}^{\text{OLS}}=YX^{⊤}{\left(XX^{⊤}\right)}^{-1}=\underset{M}{\text{argmin}}\frac{1}{2}||Y-\mathrm{MX}|{|}_{F}^{2}.$$

However, ${\hat{M}}^{\text{OLS}}$ does not guarantee nonnegative entries, so we apply the soft thresholding operator, defined by ${\left(z\right)}_{+}=\max\{z,0\}$ for z∈R, to ensure nonnegativity (Hastie *et al.* 2009). Specifically, we estimate the proportion of cell type *k* at a spot *i* by [cf Equation (3)],

(5) $${\hat{P}}_{ik}^{\text{SLS}}=\frac{{\left(\sum_{j\in \text{cell}\;\text{type}\;k}{\hat{M}}_{ij}^{\text{OLS}}\right)}_{+}}{\sum_{k}{\left(\sum_{j\in \text{cell}\;\text{type}\;k}{\hat{M}}_{ij}^{\text{OLS}}\right)}_{+}}.$$

The soft thresholding ensures all entries of the cell-type deconvolution matrix ${\hat{P}}^{\text{SLS}}$ to be nonnegative.

In conclusion, we have developed two distinct cell-type deconvolution matrix estimators, ${\hat{P}}^{\text{NNLS}}$ and ${\hat{P}}^{\text{SLS}}$. These two methods offer complementary approaches that are tailored to different computational budgets. In section below, we report our findings on the computational and statistical performance of these two estimators on benchmark ST datasets.

## 3 Software

We have developed a Python package NLSDeconv, which takes input data of all acceptable formats through *scanpy.read* function, e.g. h5ad, csv, text, h5, etc. The input data contains (i) the scRNA-seq reference data file, including the read count and metadata with a key indicating cell type, and (ii) the ST data file, including the read count and metadata with spatial location *x* and *y* coordinates as columns.

### 3.1 Preprocess

NLSDeconv provides a data preprocessing function step for scRNA-seq data. The class *Preprocessing()* requires scRNA-seq read count, ST read count, and the cell-type key for scRNA-seq data. It performs three ordered steps: (i) normalize each cell by total counts over all genes (*cellcount_norm*) (default option is True), and (ii) remove cell types and their corresponding cells if the number of cells in the cell type is less than a certain number (*cellcount_min*) (default number is 2), and (iii) perform differential expression analysis between each cell type VS. the rest, and a certain number (*gene_top*, default number is 200) of top differentially expressed genes are selected from each comparison, the union of which will form the marker gene set (Biancalani *et al.* 2021). The outputs are the preprocessed ST data and scRNA-seq data.

### 3.2 Deconvolution

Two functions of cell-type deconvolution are provided. The class *Deconv()* requires scRNA-seq read count, ST read count, and the cell-type key for scRNA-seq data.

- *.SLS()* is the command for performing SLS cell-type deconvolution. The outputs are the estimated cell-type composition matrix ${\hat{P}}^{\text{SLS}}$, algorithm running time, and list of cell types corresponding to the column name of the resulting matrix.
- *.NLS()* is the command for performing NNLS cell-type deconvolution. The required argument is learning rate (*lr*) (default is 0.1). Optional arguments are: ridge regularization parameter or list of parameters (*reg*), cross-validation fold number for list of parameters (*n_fold*), whether to use least square estimator as a warm start (*warm_start*), number of epochs (*num_epochs*), device for running the algorithm (*device*). The outputs are the estimated cell-type composition matrix ${\hat{P}}^{\text{NNLS}}$, algorithm running time, and list of cell types corresponding to the column name of the resulting matrix.

### 3.3 Visualization

We provide two functions for the visualization of deconvolution results. The required inputs are ST data and the two outputs of the previous deconvolution step: cell-type composition matrix ${\hat{P}}^{\text{SLS}}$ or ${\hat{P}}^{\text{NNLS}}$ and the list of cell types corresponding to the column name of the resulting matrix. There are optional arguments relating to display, allowing users to adjust their figures flexibly. See details in our document and tutorials.

- *overall_plt()* is the command for a spatial scatter pie plot displaying inferred cell-type composition on each location.
- *separate_plt()* is the command for spatial proportion plots of given cell types.

**Figure 1.**
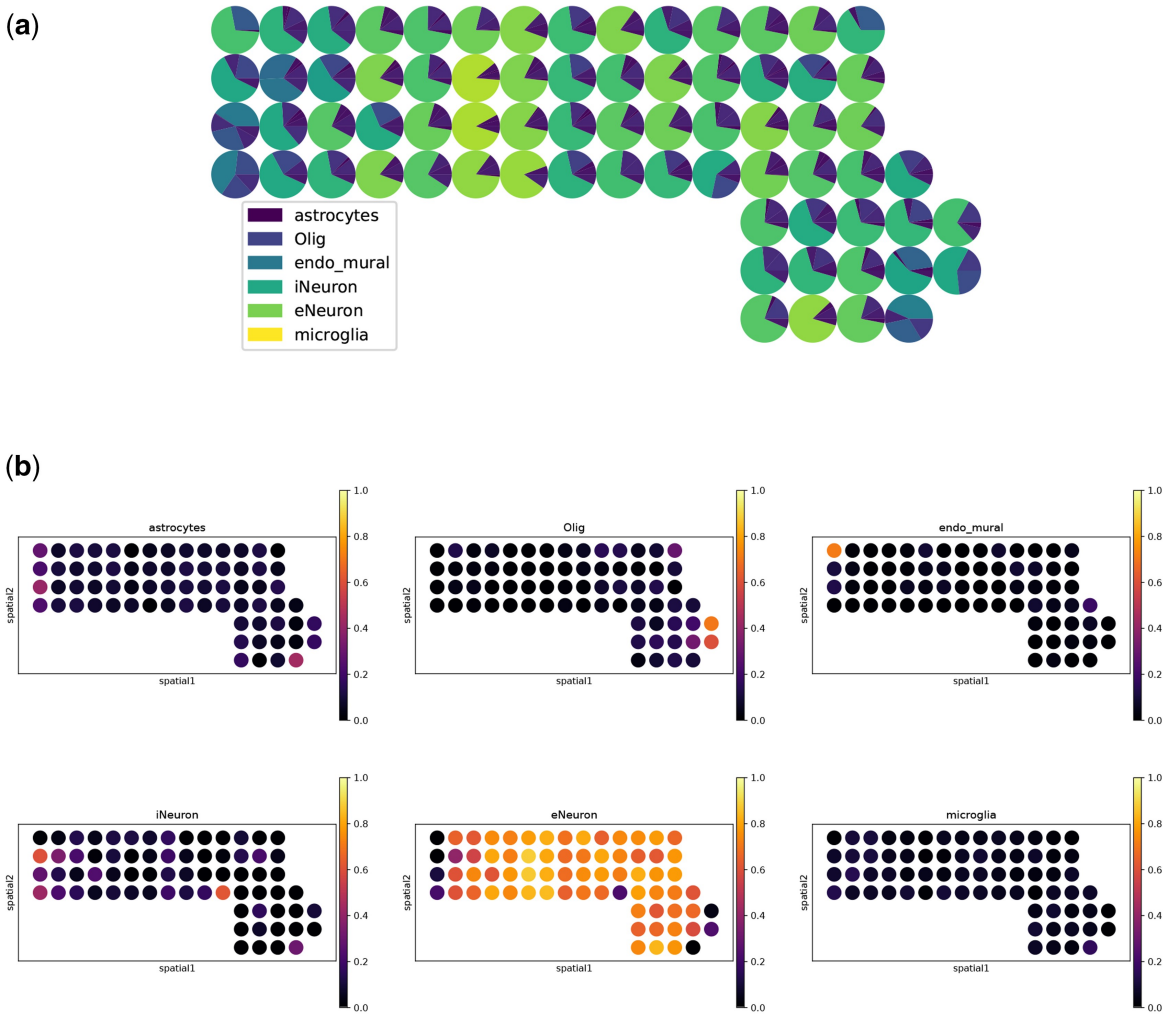
Example visualization results of SLS on the seqFISH+ (10 000) dataset. (a) A spatial scatter pie plot displays inferred cell-type composition on spatial location (produced by the command *overall_plt()*). (b) The proportion of each cell type is displayed on spatial location (produced by the command *separate_plt()*).

In Fig. 1, we show example visualization results of SLS on the seqFISH+(10 000) dataset.

## 4 Results

We assess the performance of proposed methods by comparing them with 18 existing methods on diverse benchmarking datasets reported in the recent review paper (Li *et al.* 2023). The comprehensive datasets represent two image-based platforms, seqFISH+, MERFISH, and four sequencing-based platforms: ST, 10X Visium (Visium), Slide-seqV2, and stereo-seq. For image-based datasets, as the gene expression profile is at the single-cell level and cell type labels are known, we follow the exact approach by Li *et al.* (2023) to bin the neighboring cells into spots with different resolutions and use root-mean-square error (RMSE), Jensen–Shannon divergence (JSD) to gauge the accuracy of deconvoluted cell type proportion. For the sequencing-based datasets where the true label is unknown, we also follow Li *et al.* (2023) to use the Pearson correlation coefficient (PCC) between the deconvoluted cell type proportions within each spot and the expression of the corresponding marker genes of each cell type as the performance metric. Performance of other methods are ported from the Source Data file of Li *et al.* (2023).

For SLS, it is tuning-free. For NNLS, we provide the result of two ways for selecting *λ*: NNLS(fixed) for using default λ=0.1, and NNLS(CV) for using cross-validation method to select the best *λ* from the sequence of *λ*’s, (0,0.02,0.04,…,0.2). We set the learning rate to be 0.01, the number of epochs to be 10^3^, and use a warm start. We apply the codes provided by Li *et al.* (2023) for performance measures to avoid coding bias. Tables 1 and 2 show the performance of our methods in comparison to other models on the image-based datasets with true labels. In general, our NNLS has the lowest RMSE and JSD, followed by our SLS method, which can be viewed as a fast approximation version of NNLS. For the sequencing-based datasets without true labels, to our surprise, the PCC measure suggests that SLS performs even better than NNLS, ranking first overall out of all methods (Table 3).

**Table 1.** RMSE of SLS and NNLS methods compared with 18 existing methods on image-based datasets seqFISH+ and MERFISH: a smaller RMSE indicates a more accurate method.^a^

	seqFISH+			MERFISH			
	(10 000)	(6000)	(3000)	(100)	(50)	(20)	Mean
SLS	0.109	0.113	0.126*	0.133	0.181	0.250	0.152
NNLS(fixed)	**0.104**	**0.103**	0.126*	0.109	0.165	0.243	**0.142**
NNLS(CV)	**0.105**	**0.100**	0.126*	0.108	0.161	0.234	**0.139**
dtangle	0.201	0.209	0.202	0.331	0.367	0.429	0.290
Berglund	0.272	0.267	0.280	0.177	0.177	0.286	0.243
CARD	0.126	0.129	0.141	0.188	0.188	0.264	0.172
Cell2location	0.242	0.243	0.240	0.109	**0.109**	0.247	0.198
DestVI	0.272	0.282	0.300	0.117	0.117	0.219	0.218
DSTG	0.227	0.260	0.292	0.231	0.231	0.311	0.259
NMFreg	0.296	0.291	0.291	0.180	0.180	0.310	0.258
RCTD	0.125	0.130	0.131	0.184	0.184	0.278	0.172
SD2	0.179	0.206	0.209	0.210	0.210	0.345	0.227
SpatialDecon	0.202	0.204	0.201	0.217	0.217	0.306	0.225
SpatialDWLS	0.112	0.109	**0.121**	0.213	0.213	0.525	0.215
SpiceMix	0.189	0.249	0.457	0.390	0.390	0.391	0.345
SPOTlight	0.262	0.257	0.256	0.169	0.169	0.318	0.239
STdeconvolve	0.343	0.325	0.326	0.220	0.220	0.247	0.280
stereoscope	0.223	0.226	0.228	0.193	0.193	0.274	0.223
STRIDE	0.260	0.273	0.232	0.124	0.124	0.372	0.231
Tangram	0.251	0.251	0.259	0.177	0.177	0.278	0.232
SpaOTsc	0.226	0.223	0.226	**0.104**	**0.115**	**0.151**	0.174
novoSpaRc	0.227	0.227	0.225	**0.096**	0.124	**0.162**	0.177

a The numbers in the parenthesis of seqFISH+ indicate the numbers of genes that were randomly chosen in the dataset; those of MERFISH indicate the binning sizes (in μm) for generating true labels. The last column is the mean of RMSE on all datasets. The top two methods are labeled in bold font. * means equal RMSE when rounding to three decimal places.

**Table 2.** JSD of SLS and NNLS methods compared with 18 existing methods on image-based datasets seqFISH+ and MERFISH: a smaller JSD indicates a more accurate method.^a^

	seqFISH+			MERFISH			
	(10 000)	(6000)	(3000)	(100)	(50)	(20)	Mean
SLS	0.117	**0.097**	0.118*	0.093	0.185	0.296	0.151
NNLS(fixed)	**0.098**	0.107	0.129	**0.078**	0.172	0.270	**0.142**
NNLS(CV)	0.100	0.102	0.118*	**0.077**	0.168	0.251	**0.136**
dtangle	0.148	0.156	0.142	0.475	0.508	0.655	0.347
Berglund	0.380	0.373	0.425	0.169	0.169	0.172	0.281
CARD	0.142	0.140	0.157	0.225	0.225	0.319	0.201
Cell2location	0.324	0.331	0.319	0.082	**0.082**	0.300	0.240
DestVI	0.443	0.474	0.519	0.089	0.089	**0.172**	0.298
DSTG	0.310	0.363	0.429	0.258	0.258	0.353	0.329
NMFreg	0.489	0.478	0.479	0.185	0.185	0.457	0.379
RCTD	0.143	0.145	0.140	0.156	0.156	0.242	0.164
SD2	0.216	0.261	0.277	0.221	0.221	0.528	0.288
SpatialDecon	0.275	0.279	0.270	0.236	0.236	0.282	0.263
SpatialDWLS	**0.064**	**0.073**	**0.096**	0.215	0.215	1.000	0.277
SpiceMix	0.241	0.352	0.827	0.687	0.687	0.473	0.545
SPOTlight	0.387	0.394	0.386	0.177	0.177	0.498	0.336
STdeconvolve	0.544	0.477	0.516	0.267	0.267	**0.065**	0.356
stereoscope	0.270	0.260	0.263	0.189	0.189	0.213	0.231
STRIDE	0.363	0.401	0.315	0.088	**0.088**	0.659	0.319
Tangram	0.364	0.356	0.372	0.141	0.141	0.319	0.282
SpaOTsc	0.648	0.638	0.654	0.167	0.268	0.462	0.473
novoSpaRc	0.647	0.626	0.633	0.233	0.352	0.491	0.497

a The numbers in the parenthesis of seqFISH+ indicate the numbers of genes that were randomly chosen in the dataset; those of MERFISH indicate the binning sizes (in μm) for generating true labels. The last column shows the mean JSD on all datasets. The top two methods are labeled in bold font. * means equal RMSE when rounding to three decimal places.

**Table 3.** Mean PCC of pairs of deconvoluted proportion of cell types and their corresponding marker genes from SLS and NNLS methods in comparison with 18 existing methods on sequencing-based datasets ST, Visium, Slide-seqV2, and stereo-seq: a larger PCC indicates a more accurate method.^a^

	ST	Visium	Slide-seqV2		stereo-seq		
			(11)	(17)	(gene)	(spot)	Mean
SLS	**0.241**	0.732	**0.345**	**0.333**	0.392	0.303	**0.391**
NNLS(fixed)	0.136	0.503	0.226	0.190	0.244	0.470	0.295
NNLS(CV)	0.164	0.514	**0.394**	0.286	0.322	0.472	0.359
Berglund	0.063	0.553	0.195	0.195	0.520	0.026	0.259
CARD	0.117	0.730	0.250	0.200	0.233	**0.471**	0.333
Cell2location	0.209	**0.793**	0.305	0.325	0.593	0.018	0.374
DestVI	0.081	0.642	−0.055	−0.070	0.537	0.449	0.264
DSTG	0.038	0.661	0.110	0.103	0.380	0.236	0.255
NMFreg	−0.103	0.496	0.190	0.243	0.533	0.400	0.293
novoSpaRc	−0.245	0.069	0.180	0.285	0.100	0.335	0.121
RCTD	0.160	0.011	0.305	0.320	**0.613**	0.462	0.312
SD2	0.033	0.585	0.075	0.100	0.410	0.149	0.225
SpaOTsc	−0.009	0.590	0.150	0.098	0.233	0.263	0.221
SpatialDecon	**0.295**	0.018	0.320	0.325	0.450	**0.506**	0.319
SpatialDWLS	−0.103	−0.008	−0.060	−0.025	−0.070	−0.021	−0.048
SpiceMix	−0.055	0.540	−0.065	−0.040	0.573	0.273	0.204
SPOTlight	0.125	0.750	0.270	0.233	0.360	0.433	0.362
STdeconvolve	−0.030	**0.760**	0.300	0.213	**0.603**	0.133	0.330
stereoscope	0.146	0.668	0.260	0.255	0.527	0.436	0.382
STRIDE	−0.062	0.642	0.235	0.205	0.543	0.434	0.333
Tangram	0.071	0.743	0.290	**0.365**	0.510	0.360	**0.390**

a The numbers in the parenthesis of Slide-seqV2 indicate the cell-type numbers of the dataset after the sub-cell types are integrated and the original dataset; the names in the parenthesis of stereo-seq indicate the original dataset and integrated subcellular-resolution dataset. The last column is the overall mean of PCC on all datasets. The top two methods are labeled in bold font.

One appealing feature of the SLS method is its computing efficiency. For a comparison, we pick three methods that were shown relatively more efficient in Li *et al.* (2023), including Tangram (Biancalani *et al.* 2021), RCTD (Cable *et al.* 2022), and SpatialDecon (Danaher *et al.* 2022). We consider benchmarking on two example datasets: seqFISH+ (10 000), which represents datasets with large gene numbers and small spot numbers, and MERFISH (20), which represents datasets with large spot numbers and small gene numbers. We run SLS and the other three methods on a cluster node with Intel(R) Xeon(R) Gold 6338 CPU @ 2.0 GHz and 256 GB DDR4 2666 MHz Memory with allocation of 1 CPU core and 100 GB memory. We run NNLS on Google Colab T4 GPU. The computing time is shown in Table 4. We note that Tangram is also computationally efficient. Nevertheless, its performance is less competitive. In conclusion, we recommend SLS for users with limited computation resources and NNLS for those with high accuracy standards and a demand for code flexibility.

**Table 4.** Running time (seconds) on seqFISH+(10000) and MERFISH(20) datasets.^a^

	seqFISH+	MERFISH
	(10 000)	(20)
SLS	**2.1**	**6.5**
NNLS(fixed)	48.9	**17.2**
NNLS(CV)	59.8	2496.7
Tangram	**4.0**	56.3
RCTD	87.0	1178.5
SpatialDecon	10.4	92.4

a We show the top two methods in bold.

## Data Availability

Example data of seqFISH+ available on: https://github.com/tinachentc/NLSDeconv. All the benchmark datasets are available on: https://zenodo.org/records/7674290.
